# A silicon singlet–triplet qubit driven by spin-valley coupling

**DOI:** 10.1038/s41467-022-28302-y

**Published:** 2022-02-02

**Authors:** Ryan M. Jock, N. Tobias Jacobson, Martin Rudolph, Daniel R. Ward, Malcolm S. Carroll, Dwight R. Luhman

**Affiliations:** 1 Sandia National Laboratories, Albuquerque, NM 87185 USA; 2 Center for Computing Research, Sandia National Laboratories, Albuquerque, NM 87185 USA; 3 Present Address: HRL Laboratories, LLC, Malibu, CA 90265 USA; 4 Present Address: IBM Quantum, Yorktown Heights, NY 10598 USA

**Keywords:** Quantum information, Qubits

## Abstract

Spin–orbit effects, inherent to electrons confined in quantum dots at a silicon heterointerface, provide a means to control electron spin qubits without the added complexity of on-chip, nanofabricated micromagnets or nearby coplanar striplines. Here, we demonstrate a singlet–triplet qubit operating mode that can drive qubit evolution at frequencies in excess of 200 MHz. This approach offers a means to electrically turn on and off fast control, while providing high logic gate orthogonality and long qubit dephasing times. We utilize this operational mode for dynamical decoupling experiments to probe the charge noise power spectrum in a silicon metal-oxide-semiconductor double quantum dot. In addition, we assess qubit frequency drift over longer timescales to capture low-frequency noise. We present the charge noise power spectral density up to 3 MHz, which exhibits a 1/*f*^*α*^ dependence consistent with *α* ~ 0.7, over 9 orders of magnitude in noise frequency.

## Abstract (web summary)

Spin-orbit coupling in gate-defined quantum dots in silicon metal-oxide semiconductors provides a promising route for electrical control of spin qubits. Here, the authors demonstrate that intervalley spin–orbit interaction enables fast singlet–triplet qubit rotations in this platform, at frequencies exceeding 200MHz.

## Introduction

Qubits based on the spins of electrons confined to gate-defined quantum dots (QDs) in silicon metal-oxide-semiconductor (MOS) structures have developed into a promising platform for quantum information processing. High-quality single-qubit^1,2^ and two-qubit gates^3–5^ have been demonstrated, and device manufacture is generally compatible with available silicon microelectronics fabrication methods. Qubit control techniques demonstrated in silicon MOS have utilized electron spin resonance (ESR) with microwave strip-lines^1,2,6^, electric dipole spin resonance (EDSR) using micromagnets^5^ or the intrinsic spin–orbit coupling (SOC) at the Si/SiO_2_ interface^7–9^. Making use of interfacial SOC has the appeal of driving qubit evolution with electrical-only control without reliance on the added fabrication constraints of micromagnets or on-chip microwave strip-lines.

Confining electrons to quantum dots at the Si/SiO_2_ interface has been shown to produce spin–orbit coupling that is stronger than that of bulk Si^7,9–12^. Recent observations have demonstrated that the broken crystal symmetry at the silicon heterointerface and interactions with excited valley states lead to this enhanced SOC. These effects contribute to variation of the *g*-factor in QDs^7,9–13^. The *g*-factor difference between neighboring QDs has proven to be a valuable resource, able to drive the evolution of spin qubits encoded into a singlet–triplet subspace^7,9,14^. Spin-valley coupling is known to enhance electron spin relaxation (shorter spin T_1_) near the hot spot when the valley splitting, Δ_v_, is comparable to the electronic Zeeman splitting, *E*_*Z*_ = *gμ*_B_*B*, in a QD, where *μ*_B_ is the Bohr magneton and *B* is the applied external magnetic field^15–18^. This enhanced relaxation mechanism has been used to study valley splitting^15,17,18^ and intervalley spin–orbit coupling in silicon QD devices^15,18,19^. Additionally, spin-valley coupling has been proposed as a mechanism to coherently control electron spins in silicon QDs^20–22^. This could potentially provide an intrinsic qubit control mechanism without the added fabrication complexity of integrated features such microwave striplines and micromagnets. However, coherent qubit control using spin-valley coupling has yet to be experimentally demonstrated in a silicon spin qubit.

In this work, we utilize the intervalley spin–orbit interaction near the spin-valley hot spot in a silicon MOS QD and demonstrate the ability to drive singlet–triplet rotations in excess of 200 MHz using the intervalley spin–orbit interaction. We exploit these fast rotations near the hot spot to enable unique qubit operation with high-speed all-electrical modulation between qubit logic gates and high orthogonality of control axes through electrical control of the valley splitting. These fundamental measurements establish this qubit as a candidate for future quantum information processing systems. We take advantage of this operating mode to investigate the charge noise power spectral density (PSD) in this device. We use the noise filtering properties of a Carr-Purcell-Meiboom-Gill (CPMG) dynamical decoupling pulse sequence to decouple the qubit from charge noise experienced during the spin-spin exchange interaction. Combined with long-timescale measurements of drift in the frequency of exchange driven ST qubit rotations, we find that charge noise in this device exhibits a power spectral density consistent with *S*(*f*) ~ *f*^−0.7^ over 9 decades of frequency.

## Results

### Intervalley spin–orbit interaction

**Fig. 1:**
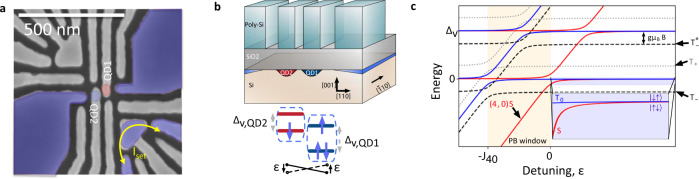
MOS DQD singlet–triplet qubit device. **a** Scanning electron micrograph of the gate structure of a device similar to that measured. The shaded regions indicate estimated areas of electron accumulation. The red and blue circles represent the locations of QD1 and QD2, respectively. We sense QD charge state transitions using a nearby single electron transistor (SET) in the lower right corner. **b** Schematic lateral view of the device structure and representation of the electron spin filling in each QD. Δ_v,QD1(QD2)_ is the valley splitting in QD1 (QD2) and *ϵ* is the QD-QD detuning. **c** Energy level diagram of the singlet–triplet system in the DQD. Δ_v_ is the valley splitting and *gμ*_B_*B* is the electronic Zeeman splitting, where g is the electron g-factor, *μ*_B_ is the Bohr magneton and *B* is the applied external magnetic field. The orange region represents the Pauli blockade window, with the singlet–triplet splitting in the (N_QD1_,N_QD2_) = (4,0) charge region denoted by *J*_40_. (inset) Energy level diagram of the *m* = 0 qubit subspace in the (N_QD1_,N_QD2_) = (3,1) charge region.

The silicon MOS double-quantum dot (DQD) used in this work is illustrated in Fig. 1a. We operate the device near the (N_QD1_,N_QD2_) = (4,0)-(3,1) charge transition. Two electrons on QD1 form a spin paired closed shell^23–25^. The interaction between the remaining two electrons is electrically controlled via the detuning bias, *ϵ*, between the QDs. For shallow detuning, there is significant electronic wave function overlap between the two electrons and the exchange energy, *J*(*ϵ*), is the dominant interaction. When the two electrons are well separated in the deep tuning regime, *J*(*ϵ*) is small and the dominant interaction is set by the interfacial SOC, which results in distinct Zeeman energies in each QD^7,9,11–13,26^.

**Fig. 2:**
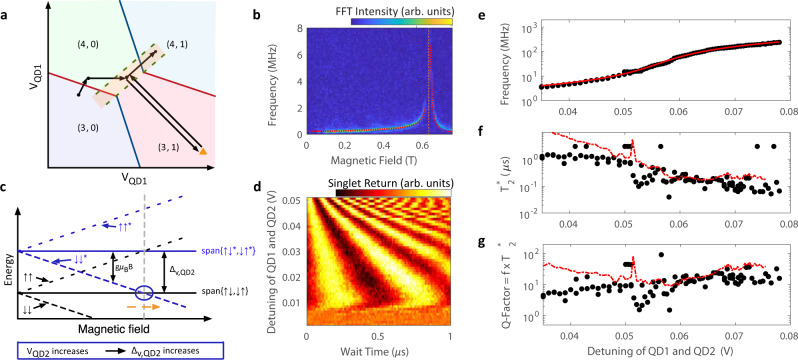
Spin-valley driven singlet–triplet rotations. **a** Schematic of the pulse sequence used to interrogate the magnetic field and detuning voltage dependencies of intervalley spin–orbit driven singlet–triplet rotations. The charge occupation of the DQD is labeled (N_QD1_,N_QD2_). **b** FFT of singlet–triplet rotations at fixed detuning in (3,1) as the external magnetic field is swept along the [010] crystallographic direction, with superimposed model fit (red dotted line). The orange dashed line indicates the spin-valley hot spot. We fit an intervalley SOC strength of 0.132 ± 0.014 μeV and a valley splitting of 73.177 ± 0.033 μeV, with uncertainty reported here as 95% confidence intervals (see Supplementary Information). **c** Magnetic field dependence of the system energy levels. Δ_v,QD2_ is the valley splitting in QD2 and *gμ*_B_*B* is the electronic Zeeman splitting, where g is the electron g-factor, *μ*_B_ is the Bohr magneton and *B* is the applied external magnetic field. The orange dashed arrow illustrates the change in the hot spot critical field as the QD-QD detuning is increased. **d** Singlet–triplet rotations at a fixed magnetic field of 0.645 T as a function of QD-QD detuning voltage. **e** Measured singlet–triplet qubit rotation frequency, *f*, as a function of QD-QD detuning voltage (black circles), with superimposed model fit (red curve). We estimate a valley splitting lever arm of 46.25 ± 0.85 μeV/V (see Supplementary Information). **f** Black circles are extracted inhomogeneous dephasing times, ${T}_{2}^{* }$, as a function of QD-QD detuning. The red dashed curve is proportional to ∣*df*/*dV*∣^−1^, the expected dependence for quasi-static charge noise, where ∣*df*/*dV*∣ is found from a numerical derivative of the data in **e**. **g** The black circles are the calculated quality factor of qubit rotations ($Q=f\times {T}_{2}^{* }$) as a function of QD-QD detuning. The red dashed curve is the expected quality factor from quasi-static charge noise found from the data in **e** and the red dashed curve in **f**.

The system is initialized by loading a (4,0) singlet ground state, then quickly transferring an electron to the (3,1) charge configuration to produce a (3,1) singlet state (i.e. rapid adiabatic passage). Here, SOC in the DQD will drive rotations between (3,1) singlet and triplet states. We then rapidly return the system to the (4,0) charge sector, where Pauli spin blockade, combined with an enhanced latching mechanism^27^, is used to read out the spin state of the two-electron system in the single-triplet basis. In Fig. 2b we show the fast Fourier transform (FFT) of SOC-driven rotations as the external magnetic field is swept along the [010] crystallographic direction. For low field strengths (*B* < 0.5 T), we observe a weak increase in evolution frequency with applied magnetic field, consistent with previous experiments^7,9,12^. In this regime an intravalley spin–orbit mechanism generates a difference in effective electron *g*-factor between the two QDs which lifts the degeneracy of the *m* = 0 states, $\left|\uparrow \downarrow \right\rangle$ and $\left|\downarrow \uparrow \right\rangle$, driving rotations between (3,1)*S* and (3,1)*T*_0_^7^. As *B* is further increased, we observed an unexpected rapid rise in rotation frequency with a sharp peak near *B* = 0.64 T. As discussed further below, these fast rotations are driven by an intervalley spin–orbit interaction which involves a coupling between distinct valley states having opposite spin. The peak position corresponds to the magnetic field at which the excited valley state, ${T}_{-}^{(1)}=\left|\downarrow \! \! {\downarrow }^{(1)}\right\rangle$, crosses the ground state *m* = 0 manifold of the two-electron system, *B*_*c*,2_, as illustrated in Fig. 2c, often referred to as the spin-valley hot spot.

Previous work has studied this regime in silicon QDs through single spin relaxation rates^15–19^. Our approach of studying coherent rotations driven by the intervalley spin–orbit interaction yields new insight into the intervalley spin–orbit interaction and its dependence on applied magnetic field. Here we provide an intuitive three-level picture of the system that describes the physics of the frequency dependence around the hot spot in Fig. 2(b) and explains how the system can be used for a qubit operating mode.

In the two-electron DQD system, the intervalley hot spot corresponds to a distortion of the *m* = 0 subspace $\{\left|\downarrow \uparrow \right\rangle ,\left|\uparrow \downarrow \right\rangle \}$ of the ST qubit due to coupling to $\left|\downarrow {\downarrow }^{(1)}\right\rangle$, the down-polarized triplet state for which the electron in QD2 is in its excited eigenvalley. This hybridizes the $\left|\downarrow \uparrow \right\rangle$ and $\left|\downarrow \!\!{\downarrow }^{(1)}\right\rangle$ states, while $\left|\uparrow \downarrow \right\rangle$ remains unperturbed. In the basis of $\left\{\left|\uparrow \downarrow \right\rangle \right.$, $\left|\downarrow \uparrow \right\rangle$, $\left.\left|\downarrow \!\!{\downarrow }^{(1)}\right\rangle \right\}$, this interaction can be represented by an effective three-level system with a Hamiltonian of the form

1 $$H=\left(\begin{array}{ccc}B\delta &0&0\\ 0&-B\delta &\gamma \\ 0&{\gamma }^{* }&{{{\Delta }}}_{{{{{{{{\rm{v,QD2}}}}}}}}}-{g}_{* }{\mu }_{B}B\end{array}\right),$$

where *δ* = *μ*_B_Δ*g*/2, with Δ*g* = *g*_1_ − *g*_2_ the difference in *g*-factors between the QDs arising from variability of interfacial SOC, *γ* is the intervalley coupling strength, Δ_v,QD2_ is the valley splitting for the QD associated with the $\left|\downarrow \!\!{\downarrow }^{(1)}\right\rangle$ state, and *μ*_B_ is the Bohr magneton. The *g*-factor governing the Zeeman shift of $\left|\downarrow \!\!{\downarrow }^{(1)}\right\rangle$ is ${g}_{* }=({g}_{1}+{g}_{2}^{(1)})/2$, the average of the *g*-factors of the ground valley of QD1 and excited valley of QD2. The eigenstates of this three-level system are

2 $$\left|+\right\rangle = {w}_{-}\left|\downarrow \uparrow \right\rangle +{w}_{+}\left|\downarrow \!\!{\downarrow }^{(1)}\right\rangle \\ \left|\uparrow \downarrow \right\rangle \\ \left|-\right\rangle ={w}_{+}\left|\downarrow \uparrow \right\rangle -{w}_{-}\left|\downarrow \!\!{\downarrow }^{(1)}\right\rangle$$

where

3 $${w}_{\pm }=\sqrt{1\pm \eta /\sqrt{{\eta }^{2}+4| \gamma {| }^{2}}}/\sqrt{2}$$

4 $$\eta ={{{\Delta }}}_{{{{{{{{\rm{v,QD2}}}}}}}}}+(\delta \,-\,{g}_{* }{\mu }_{{{{{{{{\rm{B}}}}}}}}})B,$$

with eigenenergies

5 $${E}_{\pm }\,=\,\, -B\delta +\frac{1}{2}\left(\eta \pm \sqrt{{\eta }^{2}+4| \gamma {| }^{2}}\right)\\ {E}_{\uparrow \downarrow }\,=\,\, B\delta$$

The three-level Hamiltonian has three distinct energy gaps (Δ_+_ = *E*_+_ − *E*_*↑↓*_, Δ_−_ = *E*_*↑↓*_ − *E*_−_, Δ_+−_ = *E*_+_ − *E*_−_) and, in principle, three frequencies corresponding to the rate of dynamical phase accumulation for each of these gaps could be present in the measured spectrum. However, we observe only a single rotation frequency component in Fig. 2b. This can be understood by the following physical picture. Supposing that the system is initially tuned away from the spin-valley anticrossing, the initial state prepared at the beginning of the evolution is close to $\left|S\right\rangle =\frac{1}{\sqrt{2}}(\left|\uparrow \downarrow \right\rangle -\left|\downarrow \uparrow \right\rangle )$. If the valley splitting is changed to bring the system closer to the spin-valley hot spot, the $\left|\downarrow \uparrow \right\rangle$ state adiabatically deforms into either $\left|+\right\rangle$ or $\left|-\right\rangle$. The energy gap dictating the evolution frequency is the difference between *E*_*↑↓*_ and the level (either *E*_+_ or *E*_−_) that is adiabatically connected to the initial $\left|\downarrow \uparrow \right\rangle$ state. When operating on the low-field (high-field) shoulder of the hot spot peak, the measured frequencies in Fig. 2b are dominated by rotations within the subspace spanned by $\{\left|\uparrow \downarrow \right\rangle ,\left|-\right\rangle \}$ ($\{\left|\uparrow \downarrow \right\rangle ,\left|+\right\rangle \}$), thus creating a two-level qubit system. Qubit measurement amounts to projecting back onto $\left|S\right\rangle$, with any support in the span of $\{\left|{T}_{0}\right\rangle ,\left|\downarrow \!\!{\downarrow }^{(1)}\right\rangle \}$ read out as triplet.

### Spin-valley driven singlet–triplet qubit

We realize this operating mode in the experiment by controlling the valley splitting of QD2 through modulation of the electric field^15,17,18,28^ at a constant magnetic field. We apply a field of *B* = 0.645 T along the [010] crystallographic direction, such that we are on the high magnetic field side of the hot spot peak (*gμ*_B_*B* > Δ_v_). In this case, an increase in applied electric field in QD2 will increase the valley splitting Δ_v,QD2_, shifting the location of the spin-valley hot spot to higher magnetic field. We assume a linear dependence of valley splitting as a function of gate voltage away from a reference voltage *V*_0_, ${{{\Delta }}}_{{{{{{{{\rm{v,QD2}}}}}}}}}(V)={{{\Delta }}}_{{{{{{{{\rm{v,QD2}}}}}}}}}{| }_{{V}_{0}}+{\lambda }_{{{{{{{{\rm{v}}}}}}}}}({V}_{{{{{{{{\rm{QD2}}}}}}}}}-{V}_{0})$. We refer to *λ*_v_ as the valley splitting lever arm. Since we are operating at constant magnetic field, we would expect an increase in rotation frequency in the $\{\left|\uparrow \downarrow \right\rangle ,\left|+\right\rangle \}$ subspace as we drive up the flank of the hot spot peak.

In Fig. 2d we show the singlet return signal as a function of time spent at the manipulation point in (3,1) as the QD-QD detuning, *ϵ*, is varied along Δ*V*_QD2_ = −Δ*V*_QD1_. The state is prepared in the same way as described above. For shallow detuning, we do not observe rotations since the exchange interaction, *J*(*ϵ*), is large and (3,1)S is nearly an eigenstate of the system. At moderate detuning we begin to see oscillations between singlet and triplet states due to the spin–orbit interaction, indicating a relative reduction in *J*(*ϵ*). As we pulse to deeper detuning, the voltage on the QD2 plunger increases. This enhances the vertical electric field confining QD2, resulting in an increase in valley splitting and hot spot critical field, *B*_c,2_ = Δ_v,QD2_/g_*_μ_B_, and an increase in rotation frequency (Fig. 2d). In Fig. 2e we plot the rotation frequency as a function of QD-QD detuning. Here, we demonstrate a rotation frequency in excess of 200 MHz, illustrating the ability to electrically control the intervalley spin–orbit driven frequency over a span of two orders of magnitude. Our model with an assumed linear dependence of valley splitting on gate voltage fits the data well, giving a valley splitting lever arm of 46.25 ± 0.85 μeV/V. While large-scale implementation of this qubit approach will require some level of valley uniformity, valley splittings have been shown to be tunable by a few hundred μeV in MOS devices^15,28^, which eases this constraint and bolsters the prospects for future systems.

Next, we fit the decay in oscillations of measured singlet probability as a function of wait time, *t*, for a given detuning to a Gaussian envelope, $\exp (-{(t/{T}_{2}^{* })}^{2})$. From this, we extract an inhomogeneous dephasing time, ${T}_{2}^{* }$, as a function of detuning, shown in Fig. 2f. When the interaction with the excited valley is weak, we expect the dephasing to be dominated by the hyperfine interaction with 500ppm residual ^29^Si^7,29–33^. As the interaction strength increases, the coupling to nearby electric fields will be enhanced, increasing sensitivity to charge noise. For deeper detuning, we observe a decrease in ${T}_{2}^{* }$, which follows a ${T}_{2}^{* }\propto | df/dV{| }^{-1}$ dependence, depicted as a red dashed line in Fig. 2f, which is expected for quasi-static charge noise^7,34^. At frequencies above 100 MHz (*ϵ* > 65 mV), ${T}_{2}^{* }$ is lower than the expected fit for quasi-static charge noise. The spin-valley hot spot is known to lead to an enhanced spin relaxation rate^15–19,35^, and may produce a T_1_ limited dephasing as the system is tuned closer to the *S*-${T}_{-}^{(1)}$ crossing. We estimate a lower bound for such a *T*_1_ time of no shorter 100 ns, corresponding to a relaxation rate no faster than about 10 MHz. This relaxation rate is orders of magnitude faster than measured hot spot *T*_1_ times in the literature of around 1-200 kHz^1,15,18^, but not inconsistent with extrapolated relaxation rates very close to the hot spot^18^. The quality of rotations, $Q=f\times {T}_{2}^{* }$, which compares the rotation frequency to the dephasing time, is plotted in Fig. 2g. We observe that, while the dephasing is faster at deeper detunings, the rotation frequency grows more quickly and the quality factor increases to *Q* ~ 20 at rotation frequencies above 100 MHz. We have observed hot spot driven rotation frequencies near 400 MHz in a separate ^nat^Si device, albeit with lower quality factors (see Supplementary Information). This highlights the dependence of the rotation quality on the interplay of the device tuning and the details of the intervalley coupling. Control of these parameters may provide a path to improving the rotation quality to produce higher-fidelity gate operations.

The logical basis for singlet–triplet qubits is generally represented by the linear combination of $\left|\uparrow \downarrow \right\rangle$ and $\left|\downarrow \uparrow \right\rangle$ states (e.g., $\left|S\right\rangle$ and $\left|{T}_{0}\right\rangle$). During operation, the qubit states will rotate on the Bloch sphere about the vector sum of the Z-axis governed by the exchange energy, *J*(*ϵ*), and the X-axis dictated by the difference in Zeeman splitting between the two QDs, Δ*E*_*Z*_. Logic gates are performed by electrically pulsing between regions dominated by *J*(*ϵ*) and regions dominated by Δ*E*_*Z*_. In other implementations of singlet–triplet qubits, Δ*E*_*Z*_ is fixed^24,32,36,37^. In contrast, by utilizing the electrically controlled intervalley interaction described above, we are able to independently implement high frequency spin–orbit driven gates at deep detuning, where the exchange interaction is weak, and exchange driven rotations at shallow detuning, where the intervalley interaction is weak and *J*(*ϵ*) dominates.

**Fig. 3:**
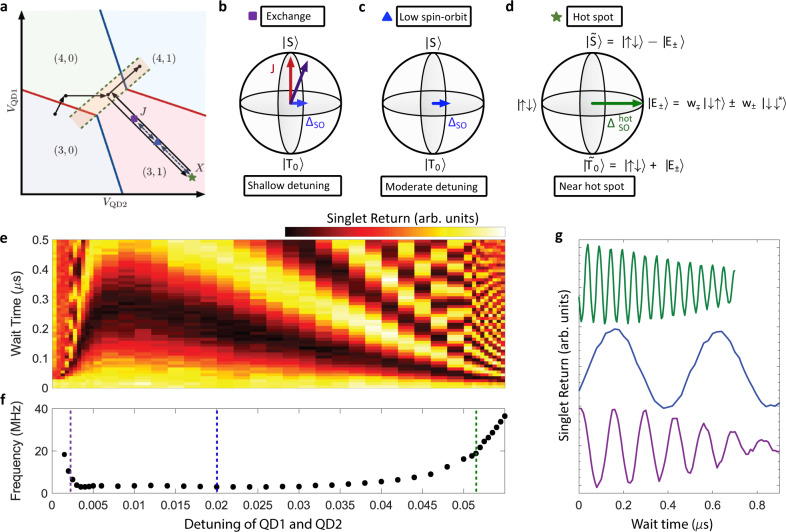
Two-axis singlet–triplet qubit control. **a** Schematic of the pulse sequence used to demonstrate all-electrical modulation between exchange-dominated and spin–orbit-dominated qubit control axes. The charge occupation of the DQD is labeled (N_QD1_,N_QD2_). Here rapid adiabatic passage is used to transfer the qubit to a (3,1)S state at moderate detuning, such that the strong spin–orbit effect is turned off, then the qubit is allowed to evolve for a *π*/2 rotation about the spin–orbit driven rotation axis. The qubit is then pulsed to some detuning point and allowed to evolve for some time, then returned to the original detuning and allowed to evolve for another *π*/2 rotation. The spin state of the qubit is then read out. The colored symbols represent three QD-QD detuning points of interest. **b**–**d** Bloch sphere representations of the qubit basis and control axes for the three QD-QD detuning points of interest. J, is the exchange interaction, Δ_SO_ is the intravalley spin–orbit interaction, and ${{{\Delta }}}_{{{{{{{{\rm{SO}}}}}}}}}^{{{{{{{{\rm{hot}}}}}}}}}$ is the intervalley hot spot interaction. **e** Singlet return probability after performing qubit control rotations as a function of QD-QD detuning voltage at a fixed magnetic field of 0.645 T. **f** Extracted qubit rotation frequency from **e**. **g** Qubit rotations for QD-QD detunings of 2.8 mV (purple), 20 mV (blue), and 55 mV (green), corresponding to exchange, low-SO, and hot spot driven qubit control, respectively.

In Fig. 3 we demonstrate simultaneous two-axis control of the intervalley driven singlet–triplet qubit. We operate on the high-field shoulder of the spin-valley hot spot and define the qubit basis in terms of the $\left|\uparrow \downarrow \right\rangle$ and $\left|+\right\rangle$ states, where

6 $$\left|\tilde{S}\right\rangle =\frac{1}{\sqrt{2}}(\left|\uparrow \downarrow \right\rangle -\left|+\right\rangle )$$

7 $$\left|\tilde{{T}_{0}}\right\rangle =\frac{1}{\sqrt{2}}(\left|\uparrow \downarrow \right\rangle +\left|+\right\rangle ),$$

with *w*_+_ ≈ 0 for shallow and moderate detuning, such that $\left|\tilde{S}\right\rangle \approx \left|S\right\rangle$ and $\left|\tilde{{T}_{0}}\right\rangle \approx \left|{T}_{0}\right\rangle$. Pulsing to shallow detuning drives exchange rotations, Fig. 3b, while for deep detunings the intervalley spin–orbit interaction is turned on, Fig. 3d. Furthermore, at moderate detuning (Fig. 3c), both the exchange and intervalley interactions are weak and spin interaction is dominated by the intravalley spin–orbit interaction^7^. This provides a regime where qubit dephasing times are limited by the hyperfine interaction with residual ^29^Si in the host lattice and decoupled from charge noise. The ability to rapidly toggle between the two control axes by pulsing to detuning regions with large (small) exchange and small (large) spin-valley coupling, respectively, provides for high-orthogonality qubit control.

We can infer a qualitative measure of orthogonality of control over this qubit from the measurements shown in Fig. 3e, f and referring to an effective qubit Hamiltonian *H* = *h*_*z*_*σ*_*z*_ + *h*_*x*_*σ*_*x*_. Since the exchange, *J*(*ϵ*), governs the *h*_*z*_ component, while the intravalley and intervalley SOC control the *h*_*x*_ component, as shown schematically in Fig. 3b–d, the relative magnitudes of *h*_*z*_ and *h*_*x*_ dictate the axis about which the qubit rotates on the Bloch sphere. For moderate detuning (middle dashed line in Fig. 3f), where the intravalley SOC contribution, Δ_SO_, dominates the *h*_*x*_ component, we observe a qubit rotation frequency of ~2 MHz. At high exchange operating point (near left dashed line in Fig. 3f), the qubit rotation frequency can reach ~20 MHz. This corresponds to *h*_*z*_/*h*_*x*_ ≈ 10. Conversely, since the exchange *J*(*ϵ*) decays quickly with detuning *ϵ*, the point of high intervalley SOC (right dashed line in Fig. 3f) corresponds to a region where the residual exchange is negligible. Here *h*_*x*_/*h*_*z*_ ≫ 1 and the axis of rotation is nearly on the equator of the Bloch sphere.

### Characterization of MOS charge noise spectrum

**Fig. 4:**
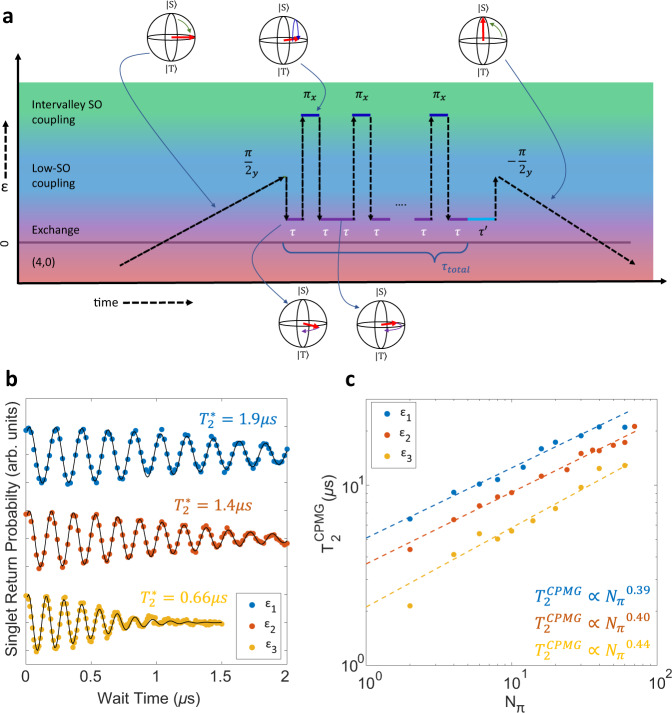
Decoupling from charge noise with CPMG. **a** Schematic for CPMG pulses. We initialize the qubit into the (4,0)S ground state and ramp adiabatically, such that the qubit transfers to the ground state (↑↓ or ↓↑) in the (3,1) charge sector at moderate detuning, away from the spin-valley hot spot. This acts as an effective *π*/2 pulse about the Y-axis of the *S*-*T*_0_ qubit basis. A fast pulse to and from a detuning, *ϵ*, where exchange is substantial, drives coherent rotations around an axis dominated by the exchange interaction. Here, charge noise drives qubit dephasing. A series of *π* pulses are then applied to decouple the qubit from charge noise. Here we operate with an intervalley spin–orbit driven rotation frequency of 20 MHz. A final wait time, $\tau ^{\prime}$, at the end of the sequence allows for the observation of the free induction decay of the refocused echo. Returning to the (4,0) charge sector adiabatically produces an effective −*π*/2 Y-pulse and projects the states onto the (4,0)S and (3,1)T_0_ basis for measurement. **b** Qubit exchange rotations at three QD-QD detuning points. Black curves are fits to oscillating Gaussian decay envelopes $\propto \exp \big({-(t/{T}_{2}^{* })}^{2}\big)$. **c** Qubit CPMG coherence time as a function of the number of refocusing pulses *N*_*π*_ for three QD-QD detuning points where exchange is the dominant spin interaction. Dashed lines are fits to the form ${T}_{2}^{{{{{{{{\rm{CPMG}}}}}}}}}\propto {N}_{\pi }^{\beta }$.

Having demonstrated high-orthogonality all-electrical control over fast Z (exchange) and X (spin–orbit) gates, we turn now to exploiting these fast operations to probe the spectral content of noise in our device at relatively high frequencies. In silicon QD based spin qubits, where magnetic noise may be reduced by the use of enriched ^28^Si, charge noise has been identified as a dominant source of error^38^. Here, charge noise may have the effect of increasing dephasing rates for one- or two-qubit gates involving the exchange interaction or when the architecture employs a magnetic field gradient from a micromagnet for spin control. CPMG pulse sequences are a well-established technique for mitigating the effects of qubit dephasing by applying a series of refocusing control pulses^39,40^ and has been successfully demonstrated with silicon spin qubits^41–45^. In Fig. 4 we demonstrate the ability to use a CPMG pulse sequence to prolong the qubit coherence time. We apply a string of intervalley spin–orbit driven pulses to decouple the qubit from charge noise during the spin-spin exchange interaction. Figure 4b shows qubit exchange rotations for three QD-QD detuning voltages. We see that for faster exchange pulses the qubit dephases more quickly, as expected for quasistatic charge noise dominated inhomogeneous dephasing in qubit exchange gates^7,34,46–48^. Figure 4c shows the CPMG coherence time, ${T}_{2}^{{{{{{{{\rm{CPMG}}}}}}}}}$, versus the number of refocusing pulses, *N*_*π*_, for the three detuning values. We observe an increase in coherence time with increasing *N*_*π*_, which follows a power-law dependence with ${T}_{2}^{{{{{{{{\rm{CPMG}}}}}}}}}\propto {N}_{\pi }^{\beta }$. We find exponents of *β* ≈ 0.39, 0.40, and 0.41 for the detuning points *ϵ*_1_, *ϵ*_2_, and *ϵ*_3_, respectively. CPMG prolongs qubit coherence by refocusing noise for timescales longer than the time between refocusing pulses. For a given total time exposed to exchange, *τ*_total_, more *N*_*π*_ pulses will decrease the time the qubit is exposed to noise before being refocused. As such, the effectiveness of CPMG to mitigate charge noise will largely be determined by the noise spectral density, *S*(*f*). For colored noise of the form *S*(*f*) ∝ *f*^−*α*^, we expect ${T}_{2}^{{{{{{{{\rm{CPMG}}}}}}}}}\propto {N}_{\pi }^{\frac{\alpha }{1+\alpha }}$^14,41–45^. A fit to the data in Fig. 4c indicates a noise spectrum with *α* ≈ 0.7.

Treating the CPMG sequence as a noise filter^49–51^ provides a noise spectroscopy method to determine the noise power spectral density (PSD). This technique has been utilized to characterize other solid-state qubits^42–45,50^. Considering the first harmonic of a bandpass filter, the strength of the noise PSD, for a given data point in Fig. 4c, is given by

8 $$S({f}_{{N}_{\pi }})=\frac{{\pi }^{2}}{4\cdot {T}_{2,{N}_{\pi }}^{{{{{{{{\rm{CPMG}}}}}}}}}},$$

where ${f}_{{N}_{\pi }}$ is the relevant noise frequency being interrogated and is given by the time between pulses when refocused echo intensity drops to 1/*e*,

9 $${f}_{{N}_{\pi }}=\frac{{N}_{\pi }}{{T}_{2,{N}_{\pi }}^{{{{{{{{\rm{CPMG}}}}}}}}}}.$$

**Fig. 5:**
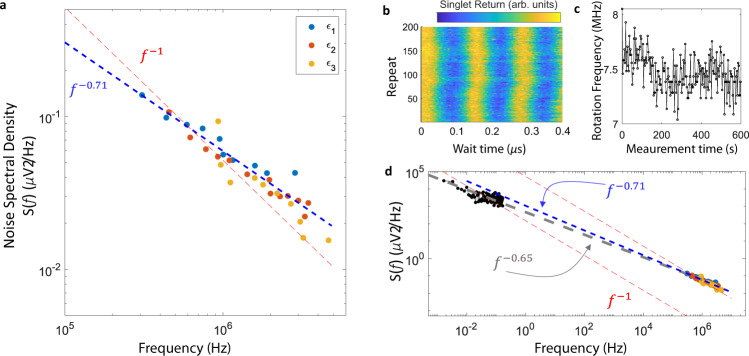
Charge noise spectrum. **a** Noise spectral density for charge noise experienced by the qubit during exchange pulses for three QD-QD detuning values. The blue dashed line is a power law fit to the data, *S*(*f*) ∝ *f*^−*α*^. The red dashed line is a fit to a 1/*f* noise spectrum as a guide to the eye. **b** Repeated experiment of singlet return probability versus wait time for an exchange pulse near detuning *ϵ*_3_ over the course of 10 min. **c** Extracted qubit frequency for data in **b** as a function of experimental measurement time. **d** combined low- and high-frequency measurements of the noise spectral density. The blue dashed line is a power law fit to the high frequency data and the gray dashed line is a power law fit to the low frequency data extracted from **c**. The red dashed lines are fits of 1/*f* spectra to the low- and high-frequency data sets, respectively, as guides to the eye.

The noise PSD is given in terms of fluctuations in exchange rotation frequency, which will be dependent on the strength of the exchange interaction at each detuning value. By using the gradient of the qubit frequency at each detuning point, *df*(*ϵ*)/*dV*(*ϵ*), we convert the spectrum to voltage noise on the QD-QD detuning, which provides a means to compare the three detuning points. The combined data are plotted in Fig. 5a, where a strong agreement in the noise PSD for all three detuning values is observed. The blue dashed line is a power law fit, which gives *S*(*f*) ∝ *f*^−0.71^.

Next, we examine the low-frequency portion of charge noise spectrum in this system. In Fig. 5b we plot the singlet return probability for repeated exchange rotation experiments near detuning *ϵ*_3_. Figure 5c shows the slow drift in the extracted exchange rotation frequency. Using a periodogram method and *df*(*ϵ*)/*dV*(*ϵ*) at this tuning, we plot the low frequency noise PSD in Fig. 5d alongside the high frequency results. A power law fit to the low frequency data (gray dashed line), extracted out to high frequency shows a *S*(*f*) ∝ *f*^−*α*^ dependence of the charge noise PSD with *α* ≈ 0.7 in the mHz to MHz frequency range. This is consistent with what is observed for single QDs in semiconductors, where the charge noise is often found to be 1/*f*-like, with *α* near 1^43–45,52–60^ and presumed to be caused by a distribution of charge fluctuators. We perform analogous measurements to characterize the power spectral density of magnetic noise and find a *S*(*f*) ∝ *f*^−1.66^ power law dependence^33,57^ (see Supplementary Information).

## Discussion

Interfacial spin–orbit interactions are known to play a significant role in the control of spin qubits in silicon QDs. In this work, we observe a rapid increase in the singlet–triplet rotation frequency near the spin-valley hot spot and develop a simple three state model to explain the observations. We utilize this effect to demonstrate an intervalley driven singlet–triplet qubit with high-orthogonality and fast electrical-only qubit control. We show the ability to electrically tune the intervalley spin–orbit interaction, enabling high-speed modulation between three qubit control regimes: (1) large exchange interaction, (2) small effective magnetic field gradient between QDs, and (3) hot spot driven qubit rotations with operational rotation frequencies exceeding 200 MHz. When the intervalley spin–orbit or exchange interactions are weak, qubit dephasing is dominated by the hyperfine interaction with the residual ^29^Si in the isotopically enriched substrate. However, for strong exchange or intervalley spin–orbit coupling, quasi-static charge noise becomes the dominant dephasing mechanism. This is the first experimental demonstration utilizing control of the spin-valley coupling to coherently drive a silicon spin qubit, establishing the intervalley driven singlet–triplet qubit as a candidate for future quantum information processing platforms.

Additionally, we highlight the utility of this qubit operating mode to probe specific physical phenomena relevant to silicon-based qubit platforms. Fits to our three-state model allow for an extraction of a valley splitting of 73.177 ± 0.033 μeV with a valley splitting lever arm of 46.25 ± 0.85 μeV/V and an intervalley SOC strength of 0.132 ± 0.014 μeV. Furthermore, we exploit the filter function properties of CPMG dynamical decoupling techniques to extract the noise power spectrum of the charge noise in this device without the added complexity of on-chip, nanofabricated micromagnets or nearby co-planar striplines that may otherwise be needed for qubit control. The fast hot spot refocusing pulses and strong coupling to charge noise when the exchange interaction is turned on allows for a probe of the noise power spectral density at high frequencies. These experiments, combined with low frequency drift measurements, reveal a noise spectrum consistent with *S*(*f*) ∝ *f*^−0.7^ for frequencies between 3 mHz and 3 MHz.

## Methods

### Device overview

The double quantum dot studied in this work was realized in a fully foundry-compatible, single-gate-layer, silicon metal-oxide-semiconductor (MOS) device structure containing an epitaxially-enriched ^28^Si layer with 500 ppm residual ^29^Si at the Si/SiO_2_ interface. The confinement and depletion gates are defined by electron beam lithography followed by selective dry etching of the poly-silicon gate layer, which produces the pattern shown in Fig. 1a. Electrons are confined at the Si/SiO_2_ interface and relevant biasing of the poly-silicon gates create quantum dot potentials under the tips of gates QD1 and QD2. The tunnel rate to the electron reservoirs under the large gates in the bottom left and top right corners of the device is controlled by the applied voltage to the reservoir gates^61^. We operate with the bottom left electron reservoir receded such that the DQD system is coupled only to the top right reservoir through QD1. A single electron transistor (SET) in the lower right corner of the device is used for charge sensing. The number of electrons in each QD is inferred from changes in current through the SET as well as by magneto- and pulsed-spectroscopy methods.

### Measurements

Measurements were performed in a ^3^He/^4^He dilution refrigerator with a base temperature of around 8 mK. The effective electron temperature in the device was 150 mK. Gates QD1 and QD2 are connected to cryogenic RC bias-T’s, which allow for the application of combined DC bias voltages and fast gate pulses. An external magnetic field is applied using a 3-axis vector magnet. We perform cryogenic preamplification of the charge sensing SET current using a heterojunction bipolar transistor (HBT)^62^.

## Supplementary information

Supplementary Information

## Data Availability

The authors declare that the data supporting the findings of this study are available within the paper and its Supplementary Information. Additional data (e.g., source data for figures) are available from the corresponding author upon reasonable request.
