# Publisher Correction: A silicon singlet–triplet qubit driven by spin-valley coupling

**DOI:** 10.1038/s41467-022-28794-8

**Published:** 2022-03-01

**Authors:** Ryan M. Jock, N. Tobias Jacobson, Martin Rudolph, Daniel R. Ward, Malcolm S. Carroll, Dwight R. Luhman

**Affiliations:** 1 Sandia National Laboratories, Albuquerque, NM 87185 USA; 2 Center for Computing Research, Sandia National Laboratories, Albuquerque, NM 87185 USA; 3 Present Address: IBM Quantum, Yorktown Heights, NY 10598 USA; 4 Present Address: HRL Laboratories, LLC, Malibu, CA 90265 USA

**Keywords:** Quantum information, Qubits

Correction to: *Nature Communications* 10.1038/s41467-022-28302-y, published online 02 February 2022.

The original version of this Article contained an error in Fig. 3, in which the blue and green vertical dashed lines were missing in panel f. The correct version of Fig. 3 is:
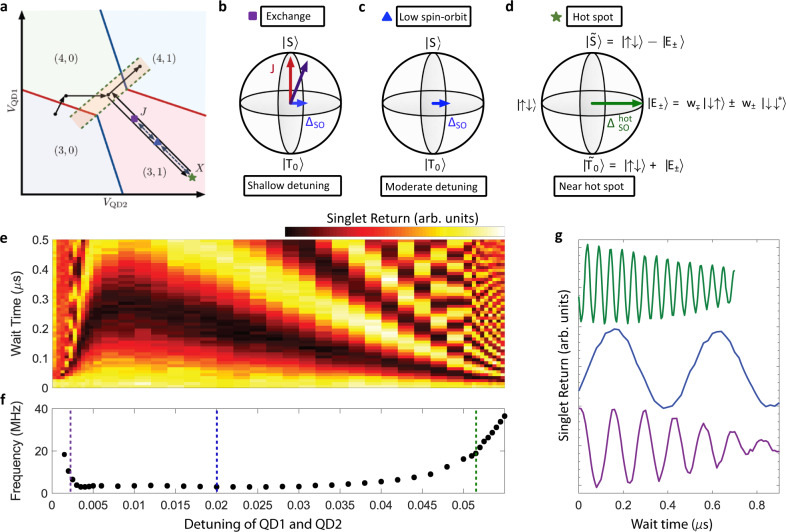


which replaces the previous incorrect version: 
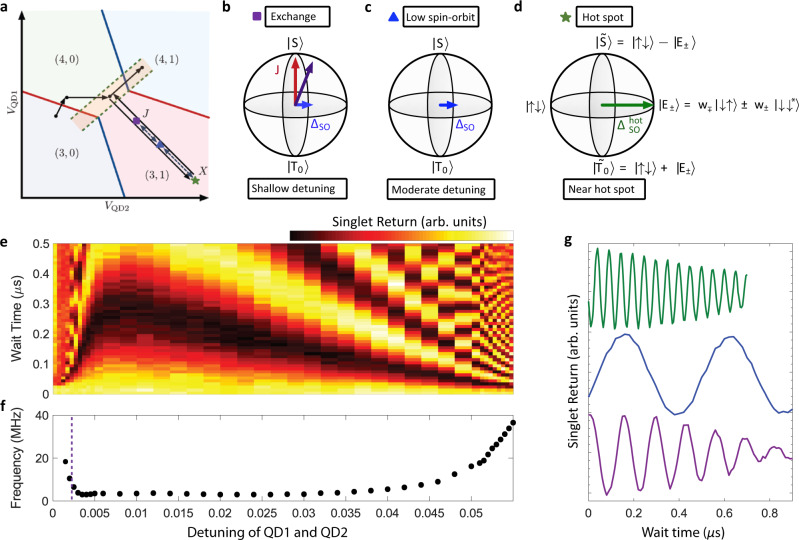


This has been corrected in both the PDF and HTML versions of the Article.

The original version of this Article contained an error in Fig. 4, in which the font on the x-axis in panel a was corrupted. The correct version of Fig. 4 is:
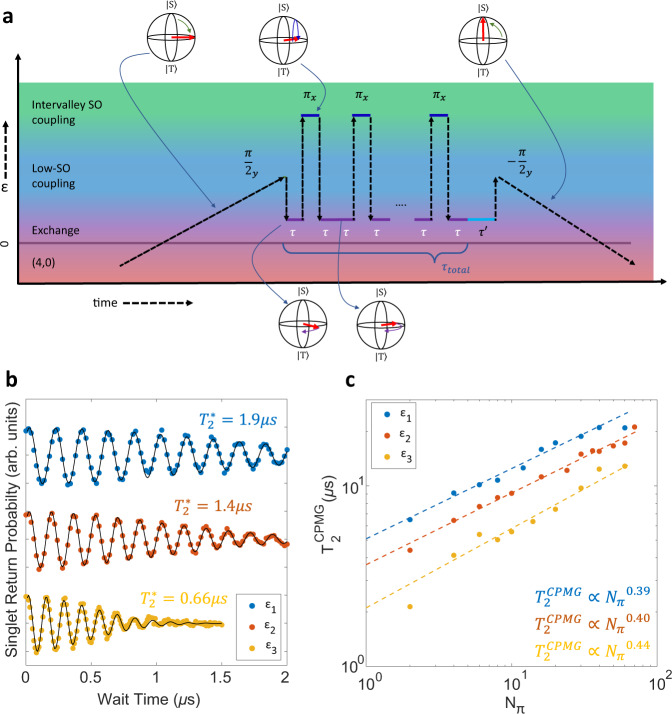


which replaces the previous incorrect version:
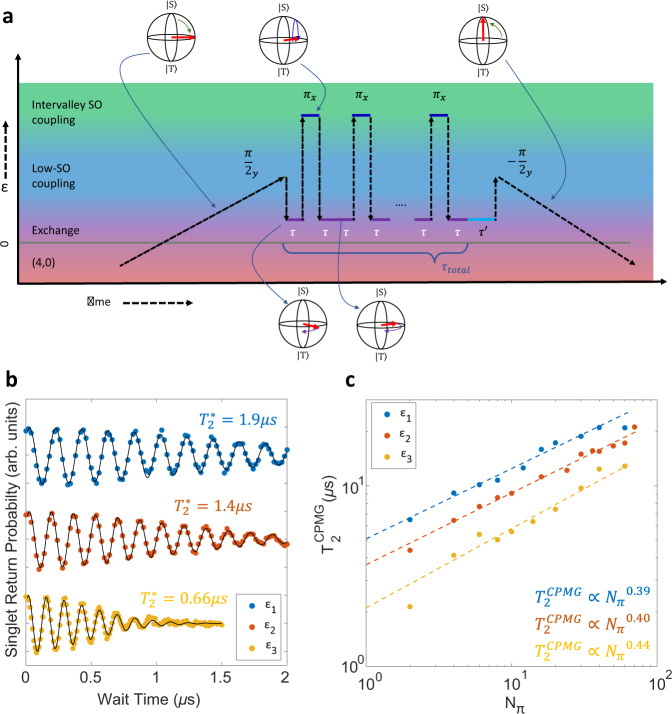


This has been corrected in both the PDF and HTML versions of the Article

In the original version of this Article, the copyright line was incorrectly given by “© The Author(s) 2022”. The correct copyright line is “© National Technology & Engineering Solutions of Sandia, LLC 2022”. This has now been corrected in the PDF and HTML versions of the Article.

