# Publisher Correction: A silicon singlet–triplet qubit driven by spin-valley coupling

**DOI:** 10.1038/s41467-022-30596-x

**Published:** 2022-05-18

**Authors:** Ryan M. Jock, N. Tobias Jacobson, Martin Rudolph, Daniel R. Ward, Malcolm S. Carroll, Dwight R. Luhman

**Affiliations:** 1 Sandia National Laboratories, Albuquerque, NM 87185 USA; 2 Center for Computing Research, Sandia National Laboratories, Albuquerque, NM 87185 USA; 3 Present Address: HRL Laboratories, LLC, Malibu, CA 90265 USA; 4 Present Address: IBM Quantum, Yorktown Heights, NY 10598 USA

**Keywords:** Quantum information, Qubits

Correction to: *Nature Communications* 10.1038/s41467-022-28302-y, published online 02 February 2022.

The original version of this Article contained an error in the author present address. The present address for Daniel R. Ward incorrectly read ‘ IBM Quantum, Yorktown Heights, NY, 10598, USA’. The present address for Malcolm S. Carroll incorrectly read ‘HRL Laboratories, LLC, Malibu, CA, 90265, USA’. This has now been corrected in both the PDF and HTML versions of the Article.

