# Supplementary material for: A silicon singlet–triplet qubit driven by spin-valley coupling
Source: Nat Commun. 2022 Feb 2;13:641. doi: 10.1038/s41467-022-28302-y (PMC8810768; doi:10.1038/s41467-022-28302-y)
Supplement: Supplementary file 1 — Supplementary Information [file 41467_2022_28302_MOESM1_ESM.pdf]

Supplementary information:
A silicon singlet-triplet qubit driven by spin-valley coupling

Ryan M. Jock,[1, *] N. Tobias Jacobson,[2] Martin Rudolph,[1] Daniel R. Ward,[1, †] Malcolm S. Carroll,[1, ‡] and Dwight R. Luhman[1]

[1]*Sandia National Laboratories, Albuquerque, NM 87185, USA*
[2]*Center for Computing Research, Sandia National Laboratories, Albuquerque, NM 87185, USA*

Measurement details

The DQD studied in this work was formed within a device nominally identical to that shown in Supplementary Figure 1(a,b). This device was fabricated in a fully foundry-compatible process using a single-gate-layer, metal-oxide-semiconductor (MOS) poly-silicon gate stack with an epitaxially-enriched ^{28}Si epi-layer with 500 ppm residual ^{29}Si. The device is operated in enhancement mode using voltage biasing of the highly doped n+ poly-silicon gates to confine electrons to quantum dot (QD) potentials under gates RD and LD (QD1 and QD2, respectively). The gates LR, RR, BLR, BRR, TLR, and TRR overlap with implanted n+ ohmic contacts regions and are biased to accumulate two-dimensional electron gas (2DEG) regions under each gate that serve as electron reservoirs for the quantum dots and single electron transistor (SET) charge sensors. The upper left and bottom right corners of the device may be used as charge-sensing SETs by confining QDs under gates TQD and BQD, respectively. In the measurements presented in the main text, we utilize only the bottom right SET.

The number of electrons in each QD may be inferred from changes in current through the SET, as depicted in Supplementary Figure 1(c). The collection of red and orange parallel lines correspond to charge transitions in QD1 and QD2, respectively. We can infer the approximate locations of QD1 and QD2 by measuring their capacitances to nearby poly-silicon gates through scans of voltages applied to pairs of poly-silicon gate electrodes. From these measurements, we obtain the relative capacitance of the QDs to each gate compared to their capacitance to RD or LD, the gates that have strongest capacitive coupling to QD1 and QD2, respectively. We plot the relative capacitances in Supplementary Figure 1(d). The measured symmetric capacitance ratios of the two QDs indicate that they are well-formed lithographic quantum dots.

We operate this system near the (N_{QD1},N_{QD2}) = (4,0)-(3,1), spin-blockaded charge anti-crossing. A charge stability diagram for the double dot system is shown in Supplementary Figure 1(c). The ground state charge configuration is determined by the detuning between dots, ϵ, which is controlled by tuning the voltages on gates RD and LD. These gates are connected to cryogenic RC bias-T's which enable the application of fast gate pulses. A schematic of the cyclical pulse sequence used in qubit measurements is shown in Supplementary Figure 2(a). The system is initialized in the (4,0) charge sector by first unloading an electron from the DQD (point U). An energy-selective pulse into the (4,0) charge state between the singlet and triplet energy levels is applied to load a (4,0)S ground state (point L). Following that, the system is plunged (point P) to a detuning ($\epsilon < 0$) close to the charge anti-crossing. The electrons are then separated (point C) and qubit manipulation pulse sequences are performed in the (3,1) charge region ($\epsilon > 0$). The system is then pulsed back to the (4,0) charge sector (point P) where, due to Pauli spin blockade, a singlet spin state is allowed to transfer to the (4,0) charge state but a triplet spin state is energetically blocked and remains in a (3,1) charge state [1]. We then use an enhanced latching mechanism for a spin-to-charge conversion (pulsing to point M)[2–8]. This technique relies on two tunneling events to load an electron onto QD2 from the reservoir, since an electron must first tunnel through QD1. This causes singlet states to remain locked in a metastable (4,0) charge state at point M, as a slow co-tunneling process is required to equilibrate. On the other hand, triplet states may quickly transfer to (4,1) by inelastic tunneling of an electron from the lead onto QD1.

CPMG analysis

The measured data for a CPMG echo experiment at detuning ϵ_3 with $N_\pi = 10$ are shown in Supplementary Figure 3(a). Here, we plot the singlet return probability for time τ' after the end of the CPMG sequence as the total qubit evolution time τ_{total} increases. The oscillations in singlet return represent the free induction decay, FID, of the refocused echo. Fitting the FID to a Gaussian envelope function for each τ_{total} gives the echo amplitude as a function of total time exposed to charge noise. Supplementary Figure 3(b) plots this as the number of refocusing pulses, N_π, is increased. As described in the main text, the relevant noise frequency being interrogated is f_{N_π}. The coherence time $T_{2,N_\pi}^{\mathrm{CPMG}}$, when the echo drops to $1/e$, indicates the noise strength near that frequency [9–12]. In Supplementary Figure 3(c) the noise power spectral density is plotted for the three detuning values shown in the main text.

Supplementary Figure 4 shows exchange dominated ST

qubit rotations, reflected in the singlet return probability as a function of time the exchange interaction is turned on for a range of QD-QD detunings. Here, we initialize the qubit into the (4,0)S ground state and ramp adiabatically into the (3,1) charge region, such that it transfers to the ground state, $|\uparrow\downarrow\rangle$ or $|\downarrow\uparrow\rangle$. A rapid pulse to and from a detuning, ϵ, where exchange is substantial drives coherent rotations around an axis depending on both exchange, $J(\epsilon)$, and the difference in Zeeman splitting, ΔE_Z. Returning to the (4, 0) charge sector adiabatically projects the states onto the (4,0)S and (3,1)T_0 basis for measurement. We then fit the rotation frequency, f, at each detuning to a smooth function to find the derivative, df/dV. This is used to convert the noise power spectral density in Supplementary Figure 3(c) from a frequency to a voltage fluctuation, which allows for a comparison of noise power at the measured detuning points shown in the main text.

Magnetic noise

We used similar techniques to characterize the power spectral density of magnetic noise in Device A. Here we look at singlet-triplet rotations at shallow detuning away from the hot spot. In Supplementary Figs. 5(b,c) we show singlet return probability for repeated experiments of SOC-driven qubit rotations and their extracted rotation frequency over the course of 20 minutes. In Supplementary Figure 5(c) we plot the noise PSD, which displays a $S(f) \propto f^{-1.66}$ power law dependence [13, 14].

Next we utilize a CPMG sequence to decouple the qubit from magnetic noise and examine the PSD at higher frequencies. To do this, we use an exchange π pulse at a qubit frequency of 8.33 MHz, illustrated in Supplementary Figure 6. When the number of refocusing pulses, N_π, is increased, we observe no change in the CPMG coherence time, T_2^{CPMG}. This suggests a flat (white) noise mechanism, which is consistent with the extracted noise power spectral density. In this low-frequency regime coherence may be limited by a T_1 qubit relaxation process, but to be definitive further studies are needed.

Supporting measurements

We independently observed valley hot spot-driven singlet-triplet rotations in another silicon device, Device B, shown in Supplementary Figure 7. This device was fabricated similarly to the device presented in the main text, but with two main differences: (1) This device has a natural silicon substrate and (2) uses a single accumulation gate SET charge sensor design [2, 8, 15] for its bottom right charge sensor. The device was operated in a similar fashion near the (N_{QD1},N_{QD2}) = (4,0)-(3,1) spin-blockaded charge anti-crossing. Supplementary Figs. 7(c,d) show the magnetic field dependence of spin-orbit driven qubit rotations as a function of magnetic field applied along the [100] crystallographic direction. The spin-valley hot spot at 0.22 T indicates a valley splitting in this device of ≈ 25 μeV. Electrical control of the hot spot driven qubit frequency is shown in Supplementary Figs. 7(e,f), where a qubit drive frequency of 400 MHz is achieved.

In Supplementary Figure 8 we show the electrical control of the qubit frequency as a function of voltage applied to QD2 for QD-QD detunings along three separate paths. The different paths behave similarly, yet show differences in the plots. This suggests that while the vertical electric field influences the valley splitting of QD2, the voltage on both the QD1 and QD2 gates modify the the intervalley spin-orbit interaction.

Valley splitting lever arm

We find that the ability to electrically modulate the intervalley SOC is consistent with control of the valley splitting, Δ_{v}, through the applied gate voltages. Since the valley splitting plays an important role in dictating the magnetic field at which the polarized triplet state $T_-^{(1)} = |\downarrow\downarrow^{(1)}\rangle$ comes into resonance with the spin state $|\downarrow\uparrow\rangle$, we can use the voltage dependence of the qubit evolution frequency in the vicinity of the hot spot to probe the variation of valley splitting with gate voltage.

To do this, we first fit to the qubit frequency versus magnetic field data of Figure 2(b) of the main text for Device A and Supplementary Figure 7(e) for Device B, respectively. We then fix these fit parameters and assume a linear variation of the valley splitting as a function of deviation of the gate voltage from the operating point at which the preceding measurements were taken,

$$\Delta_{\mathrm{v}} = \Delta_{\mathrm{v}}^0 + \lambda_{\mathrm{v}}(V - V_0) \tag{1}$$

We show fits to the model parameters in Figs. 9 and 10, with parameter estimates in Supplementary Table I. The reported uncertainties correspond to 95% confidence intervals. Note that these measurements do not permit unambiguous determination of the valley-averaged g-factor g_* in Equation 1 of the main text, so for the purpose of these parameter estimates we enforce $g_* = 2$. The results shown in Supplementary Table I are comparable with other results in silicon MOS QDs for valley splitting lever arms[16–18] and intervalley SOC strengths[16, 19, 20].

Impact of valley splitting

All silicon QD-based qubit architectures require sufficiently large valley splittings. Here, we briefly discuss a

	Device A	Device B
Intervalley SOC, γ (μeV)	0.132 ± 0.014	0.28 ± 0.05
Effective field gradient, $(10^3/h)\delta$ (MHz/T)	0.21 ± 0.15	0 ± 15
Valley splitting, Δ_v (μeV)	73.177 ± 0.033	$26.39 \pm^{0.12}_{0.14}$
Valley splitting lever arm, λ_v (μeV/V)	46.25 ± 0.85	188 ± 16
Residual exchange, J_{31} (neV)	0 ± 1.4	0 ± 60

Supplementary Table I. Model parameters for Device A (800 ppm ^{29}Si device of the main text) and Device B (^{nat}Si device providing supporting independent measurements). The reported valley splitting for each device is that of the quantum dot associated with the measured spin-valley hot spot. The valley splitting lever arms correspond to collective variation of the dot gates (QD1,QD2) by $(-V,V)$. Reported uncertainties are 95% confidence intervals based on a χ^2 analysis using the estimated linewidths. The parameter J_{31} accounts for incomplete vanishing of the exchange coupling $J(\epsilon)$ in the large positive detuning regime in which these measurements were taken.

few of the implications of valley splitting on operating a singlet-triplet qubit or ensemble of singlet-triplet qubits using this hot-spot driven control scheme.

Since the applied magnetic field is assumed to be uniform across the sample, one requirement is that the hot spot must occur at similar magnetic field values for at least one dot of any given pair of quantum dots for all double dots in the ensemble. We have demonstrated here significant electrical control over the valley splitting, as indicated in Supplementary Table I. As long as the valley splittings for all relevant dots in the device are within the range of electrical tuning, in principle the hot spot driving scheme should be accessible. Given the measured valley splitting lever arm of 46 and 188 μeV/V for the isotopically purified and natural Si devices, respectively, we can estimate roughly how much valley splitting uniformity would be required. The range of voltage values over which we routinely drive the qubit is on the order of 100 mV, which would correspond to a tunability of the valley splitting over a range of tens of μeV. Hence, a uniformity of valley splitting within this range would presumably allow for hot spot operation for all qubits in the ensemble. Valley splitting has been shown to be tunable by a few hundred μeV in other MOS QD device geometries [16, 17], which would further reduce the constraint on uniformity.

SUPPLEMENTARY REFERENCES

* Corresponding author: rmjock@sandia.gov

† Present Address: HRL Laboratories, LLC, Malibu, CA 90265

‡ Present Address: Princeton Plasma Physics Laboratory, Princeton, NJ 08543

[1] J R Petta, A C Johnson, J M Taylor, E A Laird, A Yacoby, M D Lukin, C M Marcus, M P Hanson, and A C Gossard. Coherent manipulation of coupled electron spins in semiconductor quantum dots. *Science*, 309(5744):2180–4, Sep 2005.

[2] Patrick Harvey-Collard, Benjamin D'Anjou, Martin Rudolph, N. Tobias Jacobson, Jason Dominguez, Gregory A. Ten Eyck, Joel R. Wendt, Tammy Pluym, Michael P. Lilly, William A. Coish, Michel Pioro-Ladrière, and Malcolm S. Carroll. High-fidelity single-shot readout for a spin qubit via an enhanced latching mechanism. *Phys. Rev. X*, 8:021046, May 2018.

[3] K. D. Petersson, J. R. Petta, H. Lu, and A. C. Gossard. Quantum coherence in a one-electron semiconductor charge qubit. *Phys. Rev. Lett.*, 105:246804, Dec 2010.

[4] S. A. Studenikin, J. Thorgrimson, G. C. Aers, A. Kam, P. Zawadzki, Z. R. Wasilewski, A. Bogan, and A. S. Sachrajda. Enhanced charge detection of spin qubit readout via an intermediate state. *Applied Physics Letters*, 101(23):233101, 2012.

[5] J. D. Mason, S. A. Studenikin, A. Kam, Z. R. Wasilewski, A. S. Sachrajda, and J. B. Kycia. Role of metastable charge states in a quantum-dot spin-qubit readout. *Phys. Rev. B*, 92:125434, Sep 2015.

[6] Takashi Nakajima, Matthieu R. Delbecq, Tomohiro Otsuka, Peter Stano, Shinichi Amaha, Jun Yoneda, Akito Noiri, Kento Kawasaki, Kenta Takeda, Giles Allison, Arne Ludwig, Andreas D. Wieck, Daniel Loss, and Seigo Tarucha. Robust single-shot spin measurement with 99.5% fidelity in a quantum dot array. *Phys. Rev. Lett.*, 119:017701, Jul 2017.

[7] M. A. Broome, T. F. Watson, D. Keith, S. K. Gorman, M. G. House, J. G. Keizer, S. J. Hile, W. Baker, and M. Y. Simmons. High-fidelity single-shot singlet-triplet readout of precision-placed donors in silicon. *Phys. Rev. Lett.*, 119:046802, Jul 2017.

[8] Patrick Harvey-Collard, N. Tobias Jacobson, Martin Rudolph, Jason Dominguez, Gregory A. Ten Eyck, Joel R. Wendt, Tammy Pluym, John King Gamble, Michael P. Lilly, Michel Pioro-Ladrière, and Malcolm S. Carroll. Coherent coupling between a quantum dot and a donor in silicon. *Nature Communications*, 8(1):1029, 2017.

[9] Jonas Bylander, Simon Gustavsson, Fei Yan, Fumiki Yoshihara, Khalil Harrabi, George Fitch, David G. Cory, Yasunobu Nakamura, Jaw-Shen Tsai, and William D. Oliver. Noise spectroscopy through dynamical decoupling with a superconducting flux qubit. *Nature Physics*, 7:565–570, 2011.

[10] Juha T Muhonen, Juan P Dehollain, Arne Laucht, Fay E

Hudson, Rachpon Kalra, Takeharu Sekiguchi, Kohei M Itoh, David N Jamieson, Jeffrey C McCallum, Andrew S Dzurak, et al. Storing quantum information for 30 seconds in a nanoelectronic device. *Nature Nanotechnology*, 9(12):986–991, 2014.

[11] K. W. Chan, W. Huang, C. H. Yang, J. C. C. Hwang, B. Hensen, T. Tanttu, F. E. Hudson, K. M. Itoh, A. Laucht, A. Morello, and A. S. Dzurak. Assessment of a silicon quantum dot spin qubit environment via noise spectroscopy. *Phys. Rev. Applied*, 10:044017, Oct 2018.

[12] Jun Yoneda, Kenta Takeda, Tomohiro Otsuka, Takashi Nakajima, Matthieu R. Delbecq, Giles Allison, Takumu Honda, Tetsuo Kodera, Shunri Oda, Yusuke Hoshi, Noritaka Usami, Kohei M. Itoh, and Seigo Tarucha. A quantum-dot spin qubit with coherence limited by charge noise and fidelity higher than 99.9 *Nature Nanotechnology*, 13:102, 2018.

[13] Kevin Eng, Thaddeus D. Ladd, Aaron Smith, Matthew G. Borselli, Andrey A. Kiselev, Bryan H. Fong, Kevin S. Holabird, Thomas M. Hazard, Biqin Huang, Peter W. Deelman, Ivan Milosavljevic, Adele E. Schmitz, Richard S. Ross, Mark F. Gyure, and Andrew T. Hunter. Isotopically enhanced triple-quantum-dot qubit. *Science Advances*, 1(4):e1500214, 2015.

[14] Tom Struck, Arne Hollmann, Floyd Schauer, Olexiy Fedorets, Andreas Schmidbauer, Kentarou Sawano, Helge Riemann, Nikolay V. Abrosimov, , Łukasz Cywiński, Dominique Bougeard, and Lars R. Schreiber. Low-frequency spin qubit detuning noise in highly purified 28si/sige. *arXiv:1909.11397*, 2019.

[15] L. A. Tracy, D. R. Luhman, S. M. Carr, N. C. Bishop, G. A. Ten Eyck, T. Pluym, J. R. Wendt, M. P. Lilly, and M. S. Carroll. Single shot spin readout using a cryogenic high-electron-mobility transistor amplifier at sub-kelvin temperatures. *Applied Physics Letters*, 108(6):063101, 2016.

[16] C. H. Yang, A. Rossi, R. Ruskov, N. S. Lai, F. A. Mohiyaddin, S. Lee, C. Tahan, G. Klimeck, A. Morello, and A. S. Dzurak. Spin-valley lifetimes in a silicon quantum dot with tunable valley splitting. *Nature Communications*, 4:2069, 2013.

[17] John King Gamble, Patrick Harvey-Collard, N. Tobias Jacobson, Andrew D. Baczewski, Erik Nielsen, Leon Maurer, Inès Montaño, Martin Rudolph, M. S. Carroll, C. H. Yang, A. Rossi, A. S. Dzurak, and Richard P. Muller. Valley splitting of single-electron si mos quantum dots. *Applied Physics Letters*, 109(25):253101, 2016.

[18] Xin Zhang, Rui-Zi Hu, Hai-Ou Li, Fang-Ming Jing, Yuan Zhou, Rong-Long Ma, Ming Ni, Gang Luo, Gang Cao, Gui-Lei Wang, Xuedong Hu, Hong-Wen Jiang, Guang-Can Guo, and Guo-Ping Guo. Giant anisotropy of spin relaxation and spin-valley mixing in a silicon quantum dot. *Phys. Rev. Lett.*, 124:257701, Jun 2020.

[19] Xiaojie Hao, Rusko Ruskov, Ming Xiao, Charles Tahan, and HongWen Jiang. Electron spin resonance and spin–valley physics in a silicon double quantum dot. *Nature communications*, 5(5):3860, May 2014.

[20] J. C. C. Hwang, C. H. Yang, M. Veldhorst, N. Hendrickx, M. A. Fogarty, W. Huang, F. E. Hudson, A. Morello, and A. S. Dzurak. Impact of g-factors and valleys on spin qubits in a silicon double quantum dot. *Phys. Rev. B*, 96:045302, Jul 2017.

ACKNOWLEDGMENTS

Sandia National Laboratories is a multi-mission laboratory managed and operated by National Technology and Engineering Solutions of Sandia, LLC., a wholly owned subsidiary of Honeywell International, Inc., for the U.S. Department of Energy's National Nuclear Security Administration under contract DE-NA-0003525. This paper describes objective technical results and analysis. Any subjective views or opinions that might be expressed in the paper do not necessarily represent the views of the U.S. Department of Energy or the United States Government.

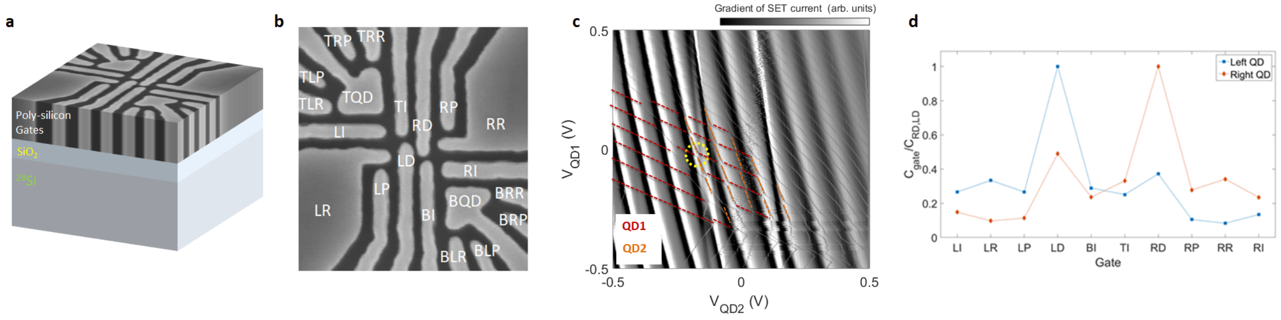

Supplementary Figure 1. MOS DQD device structure. (a) A cartoon schematic of the MOS gate stack. (b) A top-down SEM of the single-layer poly-silicon gate design with the gate names labeled in white.(c) Charge stability diagram of the DQD. Here, we plot the gradient of the SET charge sensor current as the gates QD1 and QDs are varied about fixed offset voltages ($V_{DC,QD1} = 3.4$ V and $V_{DC,QD2} = 3.9$ V). The broad diagonal background features are due to Coulomb blockade peaks of the SET charge sensor. The sharp features correspond to charge transitions in the QDs. The red and orange dashed lines are guides to the eye for QD_1 and QD_2 charge transitions. The yellow circle represents the (N_{QD1},N_{QD2}) = (4,0)-(3,1) charge region where this work was done. (d) Gate capacitance to QDs relative to LD (blue) and RD (red) gates.

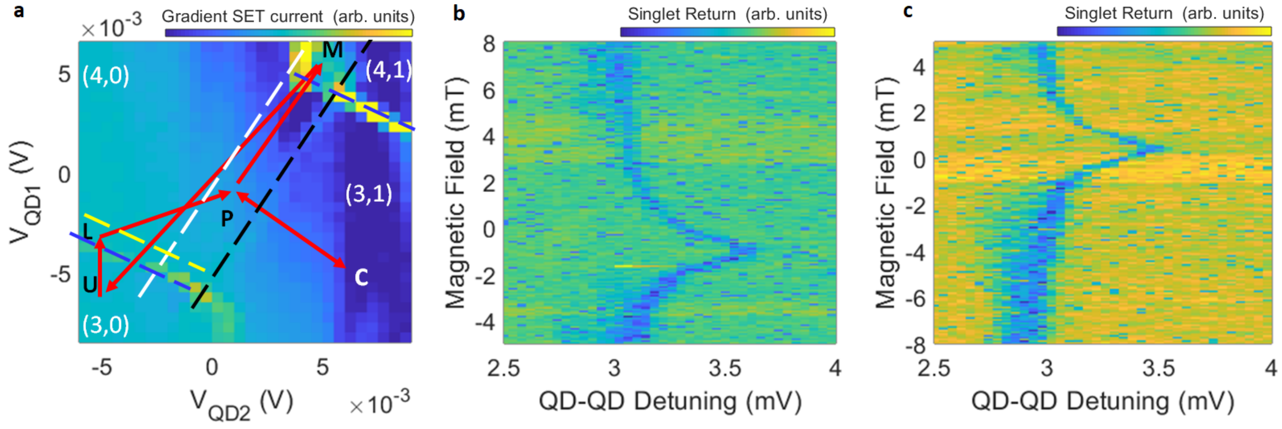

Supplementary Figure 2. Device operation and spin funnels. (a) A pulsed charge stability diagram for the (N_{QD1},N_{QD2}) = (4,0)-(3,1) anticrossing, showing the gradient of the charge sensor current. The red arrows depict a general pulse sequence for controlling the qubit: The system is initialized by first unloading an electron from the DQD (point U). An energy-selective pulse is applied to load a (4,0)S ground state (point L). The system is then plunged (point P) near the charge anti-crossing. The electrons are then separated (point C) and qubit manipulation pulse sequences are performed in the (3,1) charge region. The system is then pulsed back to point P where, due to Pauli spin blockade, a singlet spin state is allowed to transfer to the (4,0) charge state but a triplet spin state is energetically blocked and remains in a (3,1) charge state. An enhanced latching mechanism is then utilized for a spin-to-charge conversion (point M). Here the qubit control point, C, may consist of a complex voltage detuning sequence for qubit manipulation. The black and white dashed lines correspond to the location of the singlet and triplet state inter-QD charge preserving lines, respectively. (b,c) Spin funnel measurements indicating degeneracy between (3,1)S and (3,1)T_- states as a function of QD-QD detuning voltage for magnetic fields applied along the [100] and [110] in-plane crystallographic directions, respectively.

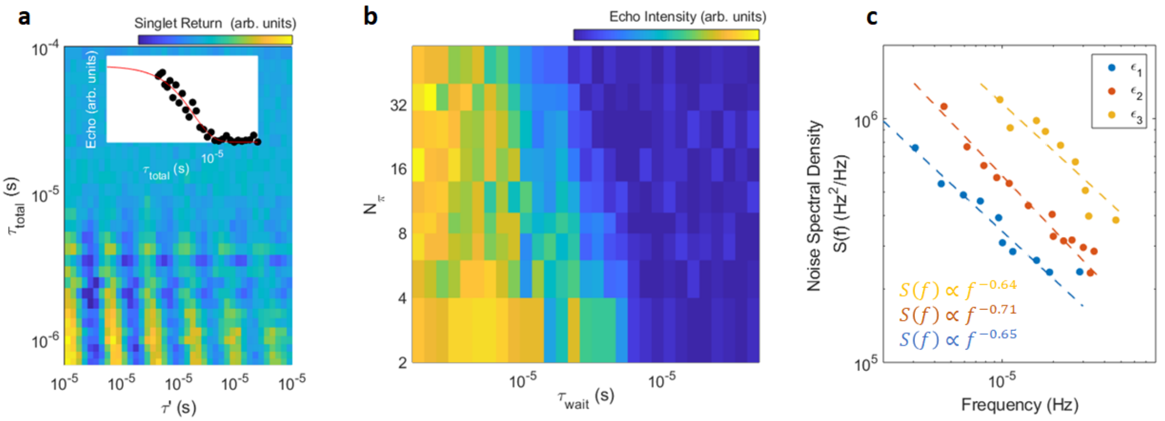

Supplementary Figure 3. CPMG experiments. (a) CPMG echo at detuning ϵ_3 with $N_\pi = 10$. We plot singlet return probability as function of wait time after the qubit is refocused, τ', as a function of τ_{total}, the total qubit manipulation time for a CPMG sequence. For each τ_{total}, the echo can be fit to an oscillating Gaussian decay to extract the echo amplitude. (a,inset) Echo amplitude as a function of total time exposed to charge noise, τ_{total}, for $N_\pi = 10$. Red line is a fit to a decay of the form $\exp(-(t/T_2^*)^n)$. (b) Echo amplitude as a function of wait time, τ_{wait}, as N_π is stepped. (c) Frequency noise spectral density for charge noise experienced by the qubit during exchange pulses for three QD-QD detuning values. The dashed lines are power law fits to the data.

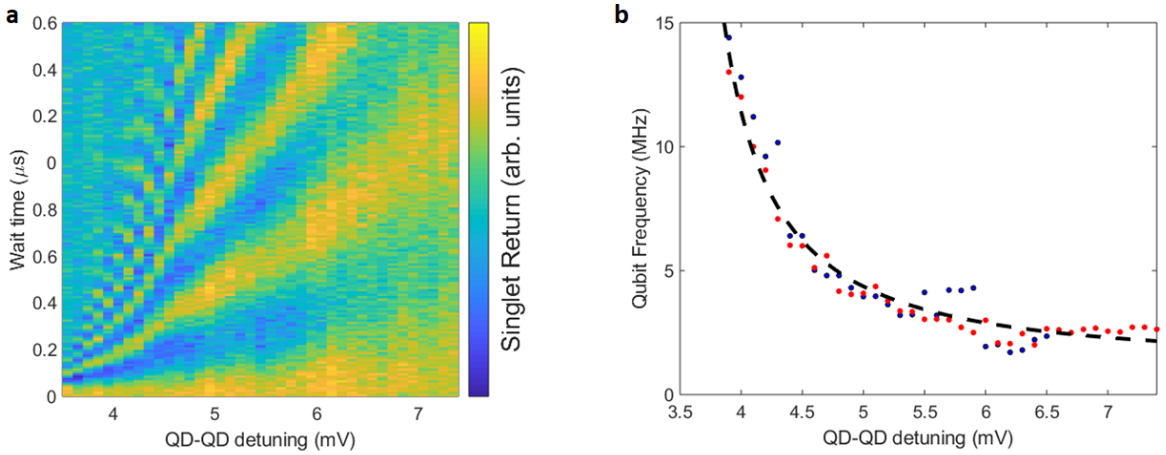

Supplementary Figure 4. DQD exchange. (a) Exchange rotations at a fixed magnetic field of 0.645 T as a function of QD-QD detuning voltage. (b) Extracted frequency of qubit exchange rotations as a function of QD-QD detuning voltage. The blue and red circles are two experimental data sets and the black dashed line is a fit to the form $f(\epsilon) = \sqrt{J(\epsilon)^2 + \Delta E_Z^2}$, with $J(\epsilon) \propto \frac{t_c^2}{\epsilon}$.

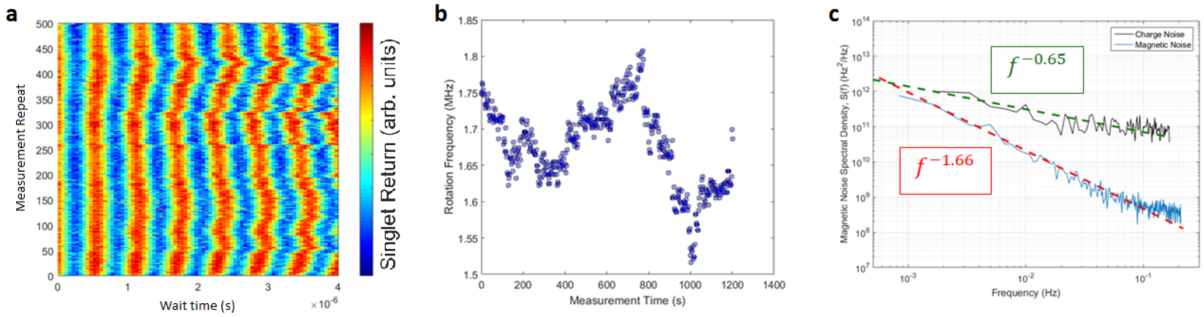

Supplementary Figure 5. Low frequency magnetic noise. (a) Repeated experiment of singlet return probability versus wait time for spin-orbit driven singlet-triplet rotations over the course of 20 minutes. (b) Extracted qubit frequency for data in (a) as a function of experimental measurement time. (c) The low frequency noise spectrum extracted using a periodogram method for magnetic (blue data) and charge (black data) noise. The red and green dashed lines are fits to $S(f) \propto f^{-\alpha}$ noise spectra.

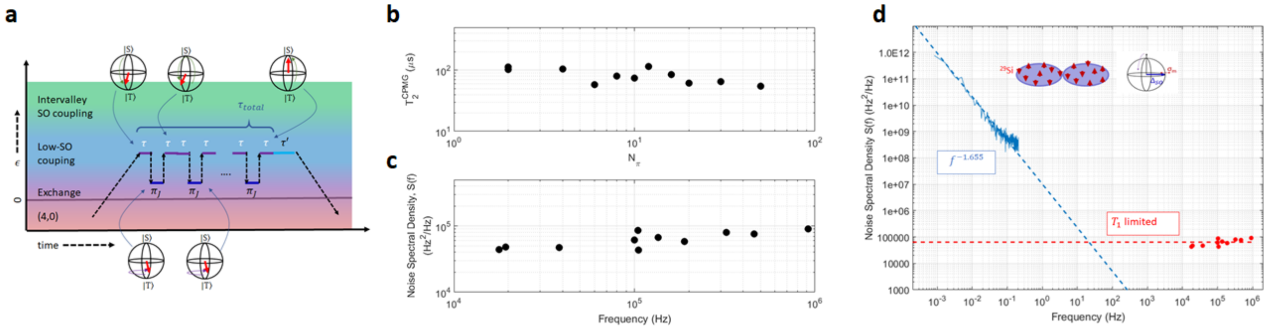

Supplementary Figure 6. Decoupling from magnetic noise with CPMG. (a) Schematic for CPMG pulses to investigate magnetic noise. We initialize the qubit into the (4,0)S ground state and transfer one electron to the neighboring dot using rapid adiabatic passage, such that the qubit remains a singlet in the (3,1) charge sector. The qubit is then allowed to evolve and dephase due to fluctuations in the Overhauser fields between the two QDs. A series of fast pulses to and from a detuning ϵ, where J is substantial, drive $\pi/2$ pulses to decouple the qubit from magnetic noise. A final wait time, τ' at the end of the sequence allows for the observation of the free induction decay of the refocused echo. Returning to the (4,0) charge sector by rapid adiabatic passage projects the states onto the (4,0)S and (3,1)T_0 basis for measurement. (b) Qubit CPMG coherence time as a function of the number of refocusing pulses, N_π. (c) High frequency noise spectrum for magnetic noise experienced by the qubit. (d) Combined low- and high-frequency measurements of the magnetic noise power spectral density. The blue and red dashed lines are fits to a power law, $S(f) \propto f^{-1.66}$, and constant (white noise), respectively. (inset) Fluctuations in the nuclear spins of the residual ^{29}Si in each QD will cause fluctuations in their respective Zeeman splittings and cause dephasing of singlet-triplet qubit rotations.

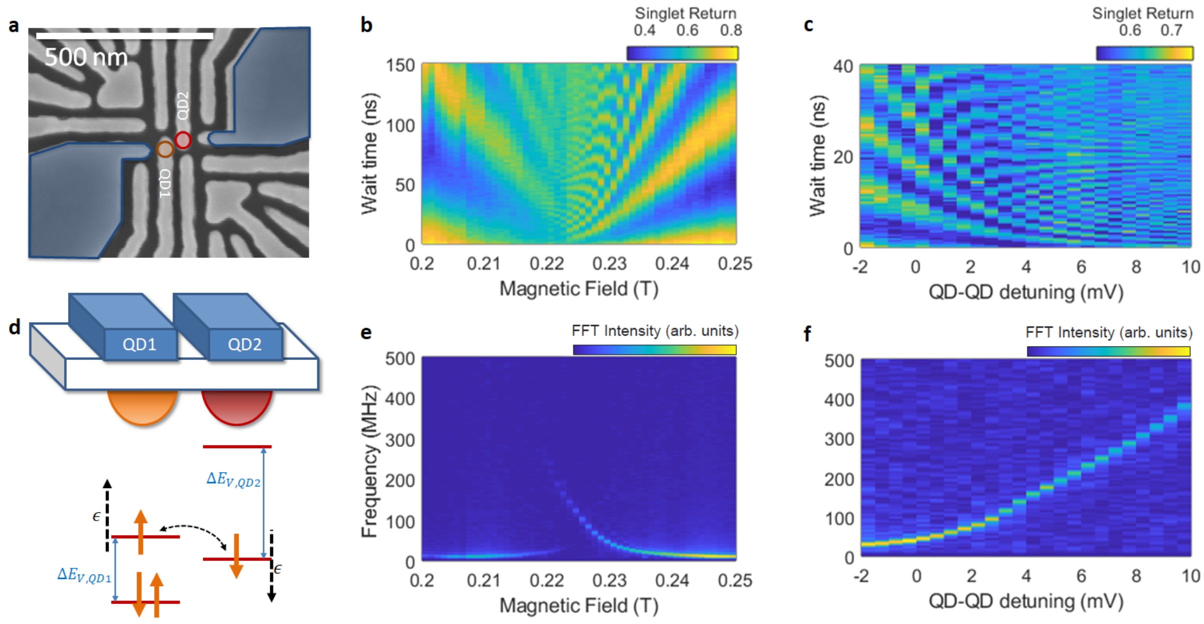

Supplementary Figure 7. Spin-valley interaction in a ^{nat}Si DQD device.(a) Scanning electron micrograph of the gate structure of a device similar to that measured. The overlaid regions indicate the estimated locations of electron accumulation (b) Change in singlet return as a function of X-rotation manipulation time as the magnetic field is varied along the [100] crystallographic direction. (c) Change in singlet return as a function of X-rotation manipulation time as the QD-QD detuning is varied. (d) A cartoon representation of the electron spin filling in each QD in this device. $\Delta E_{\mathrm{v,QD1(QD2)}}$ is the valley splitting in QD1 (QD2) and ϵ is the QD-QD detuning. (e) The FFT extracted rotation frequency as a function of magnetic field for the data in (b). (f) The FFT extracted rotation frequency as a function QD-QD detuning for the data in (c).

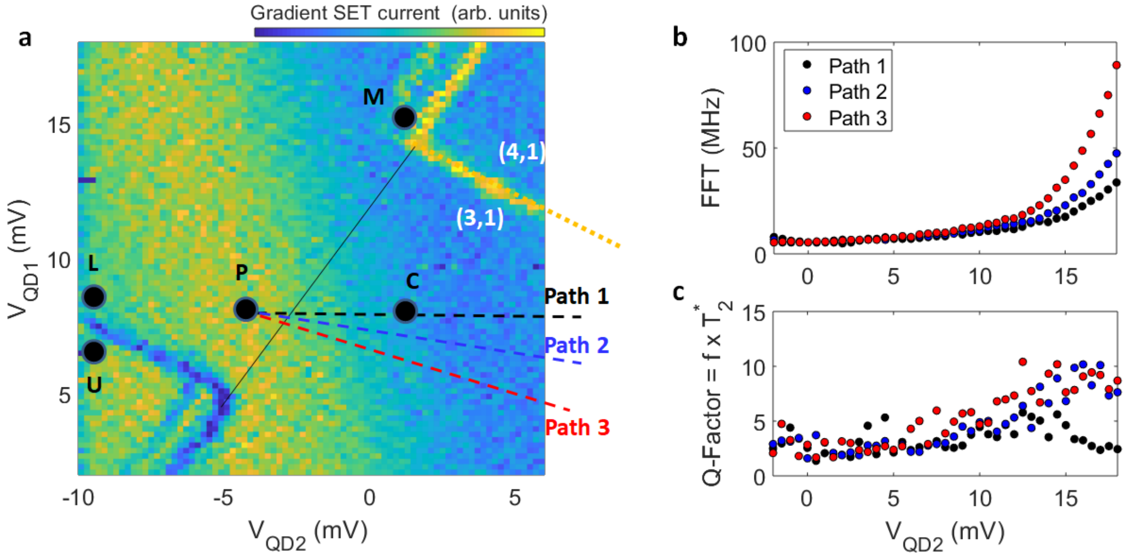

Supplementary Figure 8. DQD tuning path. (a) A pulsed charge stability diagram for the (N_{QD1},N_{QD2}) = (4,0)-(3,1) anti-crossing in Device B, showing the gradient of charge sensor current. The black circles represent the qubit reset (U), load (L), plunge (P), manipulation (C) and readout (M) points. The qubit manipulation point is varied in three experiments along three detuning paths (black, blue, and red dashed lines). (b) Extracted rotation frequency vs voltage applied to QD2 for the three paths. (c) The extracted frequency and dephasing times, T_2^*, give the Q-factors of the data for paths 1, 2, and 3.

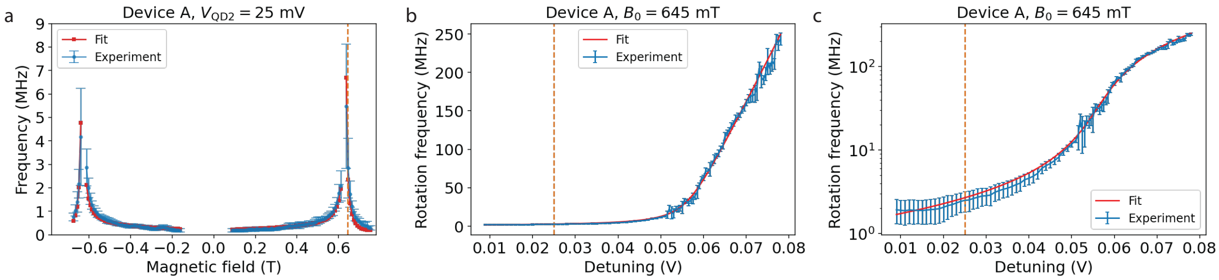

Supplementary Figure 9. Model fits for Device A. Fits to magnetic field dependence (a) and gate voltage dependence (b and c, with same data plotted with frequency on linear and logarithmic scales for clarity, respectively). The vertical dashed line in each plot corresponds to the parameter held fixed in the other plot. Error bars represent $\pm\sigma$ for Gaussian fits to linewidths.

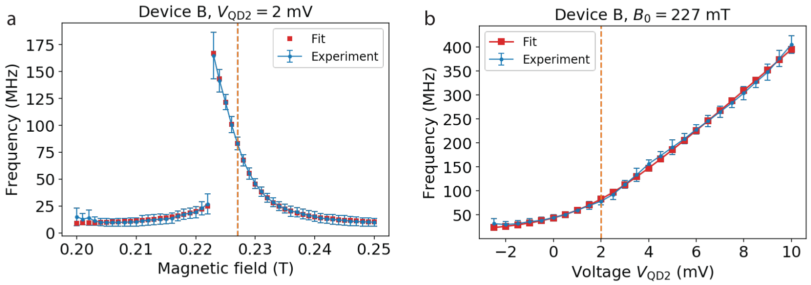

Supplementary Figure 10. Model fits for Device B. Fits to magnetic field dependence (a) and gate voltage dependence (b). The vertical dashed line in each plot corresponds to the parameter held fixed in the other plot. Error bars represent $\pm\sigma$ for Gaussian fits to linewidths.
